# Apple phenolic compounds enhance dental remineralization in human: a multifactorial *in situ* study

**DOI:** 10.1016/j.jare.2026.01.034

**Published:** 2026-01-16

**Authors:** Niklas Roeder, Antje Geiken, Vivian Schaarschmidt, Louisa Pannenbeckers, Stefan Dussling, Louise Holtmann, Julia Jensen-Kroll, Fynn Brix, Tim Hollstein, Tobias Demetrowitsch, Helen Gorges, Stanislav N. Gorb, Frank Will, Dominik M. Schulte, Mario Hasler, Ralf Schweiggert, Christof Dörfer, Karin Schwarz

**Affiliations:** a Division of Food Technology, Institute of Human Nutrition and Food Science, Kiel University, Kiel, Germany; b Clinic for Conservative Dentistry and Periodontology, Kiel University, Kiel, Germany; c Chair of Analysis and Technology of Plant-based Foods, Department of Beverage Research, Geisenheim University, Von-Lade-Strasse 1, Geisenheim 65366, Germany; d Institute of Diabetes and Clinical Metabolic Research, University Medical Center Schleswig‑Holstein, Kiel University, Düsternbrooker Weg 17, Kiel 24105, Germany; e Zoological Institute Functional Morphology and Biomechanics, Kiel University, Kiel, Germany; f Variation Statistics, Kiel University, Kiel, Germany

**Keywords:** Dental caries, Enamel demineralization, In situ model, Fruit juice, Saliva flowrate, Decay missing filled teeth (DMFT)

## Abstract

**Introduction:**

Excessive consumption of sugar-sweetened beverages is a major contributor to dental caries and a global public health concern due to its negative effects on oral health. Phenolic compounds found in edible plants have shown promising effects on caries prevention. Several studies indicate that these bioactive substances can inhibit caries formation through various mechanisms. However, studies which consider the complex interplay of the various factors are missing. In particular, human studies on the caries reducing effects of phenolic compounds in the co-presence of fermentable sugars are lacking.

**Objective:**

This human intervention study investigated the effect of daily consumption of an apple juice-based drink enriched with phenolic compounds on caries development, compared to the exact same drink without phenolic compounds.

**Methods:**

30 healthy male participants were enrolled in a double-blind, randomized, cross-over design. Caries development was measured using lesion depth in bovine tooth enamel samples installed in a removable dental appliance. A mixed model was used to analyze the data, including treatment effects, various oral parameters and interaction terms.

**Results:**

The analysis showed that consumption of the drink enriched with phenolic compounds resulted in a significant reduction in enamel lesion depth compared to the control, after adjustment for oral parameters and their interactions.

**Conclusion:**

Enrichment of phenolic compounds could be a promising strategy to mitigate the cariogenic effects of fermentable sugars in beverages. Analyzing the complex interactions between the oral parameters and treatment we bridge the translational gap of *in vitro* studies and achieve physiological relevance.

## Highlights

• Consumption of phenolic compound-enriched apple juice led to significantly less caries lesions compared to the placebo.
• Apple juice with a defined phenolic compound profile and a compositionally identical phenolic-free placebo were used.
• An individual appliance with bovine enamel samples enabled in-situ examination of demineralization and remineralization.
• A mixed statistical model was employed to consider individual dental factors that influence lesion depth.

## Graphical abstract

**Figure:**
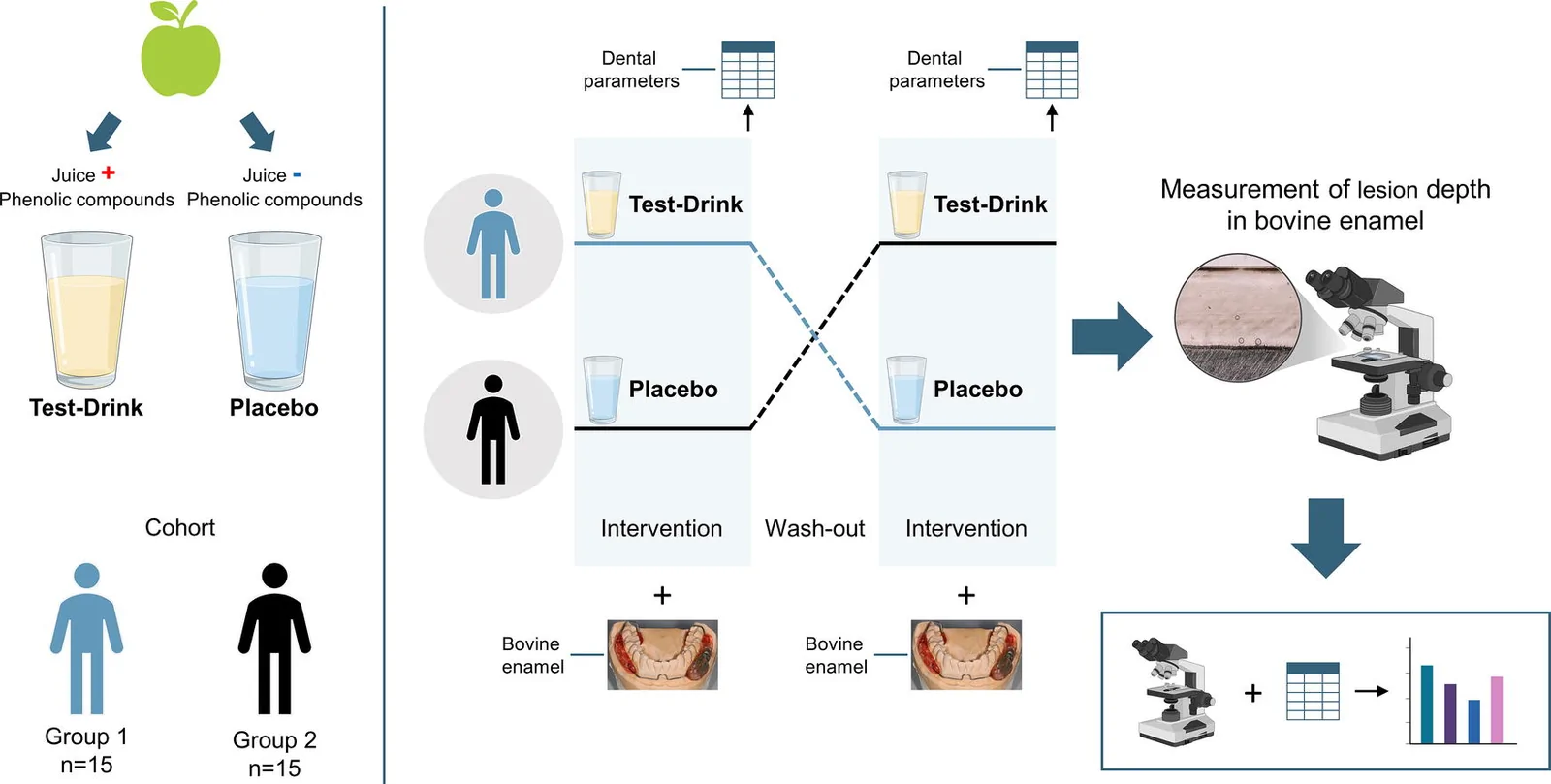

## Introduction

Dental caries is one of the most prevalent non-communicable diseases worldwide, affecting almost a third of the global population [1]. It results from a complex interplay between dietary components, microbial activity, and host factors, ultimately leading to the demineralization of tooth enamel and dentin [2]. The primary biochemical driver is acid production by oral bacteria which metabolize fermentable sugars into organic acids, thereby lowering plaque pH and dissolving tooth minerals. Although dental caries is largely preventable, its incidence is still at a high level, particularly in industrialized societies where dietary patterns include frequent consumption of sugar-rich foods and beverages [3]. The cariogenic effect of fruit juices is attributed to the natural presence of fermentable sugars. Mono- and disaccharides in juice can be metabolized by oral bacteria, leading to acid production that promote enamel demineralization [4]. Additionally, the intrinsic acidity of fruit juices can directly erode tooth enamel, weakening its structure [5], [6]. Conversely, fruit juice also contains secondary plant metabolites such as phenolic compounds, which are known for their antibacterial, antioxidant and potentially remineralizing properties [7], [8]. In this context, apples are a particularly interesting source. They have a high content of phenolic compounds and a wide structural diversity [9], and both the fruit and its juice are among the most commonly consumed fruit products worldwide [10], [11].

It is suspected that these compounds exert protective effects on oral health by reducing bacterial acid production, inhibiting biofilm formation, and limiting bacterial adhesion [12].

Although the potential oral health benefits of phenolic compounds are widely discussed, most of the available evidence originates from mechanistic or *in vitro* studies conducted under highly simplified conditions. These systems do not reflect the complexity of the human oral environment, in which factors such as saliva flow and buffer capacity, biofilm formation and dietary exposure interact continuously. Human *in situ* models that capture these real-life conditions are rare and studies lacking for sugar-containing beverages [13], [14].

The few existing *in situ* studies on phenolic compounds have focused primarily on bacterial adherence, biofilm development and pellicle ultrastructure, and have typically used phenolic compound containing rinses or isolated extracts rather than habitual dietary intake including the co-presence of fermentable sugars [12], [15].

Consequently, the preventive potential of phenolic compounds within a realistic controlled *in situ* caries study design, where sugar and acids counteracting the activity of phenolic compounds needs to be investigated.

To close this gap, we conducted a double-blind, randomized controlled human intervention study with a cross-over design (two times two weeks), involving 30 healthy male participants. We compared the anticariogenic effects of an apple juice-based drink enriched with phenolic compounds (test drink) with a placebo version from which only the phenolic compounds had been removed. This differs from other studies, which often only adjust the placebo for sugar or acid content. We investigated the cariogenic effects *in situ* using an appliance containing a sample of bovine tooth enamel, which was placed in the participant’s oral cavities. This method is considered as the gold standard for testing cariogenic effects on tooth enamel in participants oral cavities [16], [17], [18], [19]. Additionally, comprehensive data collection on relevant oral parameters and statistical analysis enable us to demonstrate the effect of phenolic compounds on enamel lesion depths in participants differing in factors such as saliva flow and buffering capacity, biofilm formation and dental health. Our approach directly quantifies mineral loss and gain in enamel lesions, providing a functional and clinically relevant measure of cariogenic activity under real-life exposure conditions.

## Material and methods

### Study design

The study was conducted in a randomized, double-blind, two-way cross-over design at the University Medical Center Schleswig-Holstein (UKSH), Kiel University, Germany. 30 healthy male participants in the age of 21–57 took part in the study. The participants had a body mass index (BMI) between 20.5–29.5 kg/m^2^. Exclusion criteria for participating in the study were metabolic or endocrine diseases, malabsorption syndrome, allergies, smoking, alcohol and drug abuse, the use of supplements, medication (such as antidepressants, antibiotics), fructose intolerance, the presence of a defibrillator or cardiac pacemaker, known allergies to the materials used, a magnetic resonance imaging (MRI) examination planned during the study period, reflux disease, bulimia and epilepsy. Further dental exclusion criteria were existing erosions or carious defects on the teeth requiring treatment, a professional tooth cleaning less than 4 weeks ago, a periodontal screening index (PSI) > 2, no stable occlusion, orthodontic therapy carried out at the time of the study, wearing an occlusal splint, and no sufficient number of teeth, i.e., no terminal molar in the fourth or third quadrant and more than 2 missing teeth per quadrant. Participants were recruited from the general population of Kiel, Germany.

They were recruited through online platforms, university channels and announcements within local community groups and organizations. A total of 157 people were interested in participating, 79 people were screened and 30 participants were ultimately included. The recruitment process is illustrated in Fig. 1. All included participants successfully completed the study. The randomization of the participants was carried out using RANDOM.ORG by a team member who was not involved in participant interaction or outcome assessment.

**Fig. 1:**
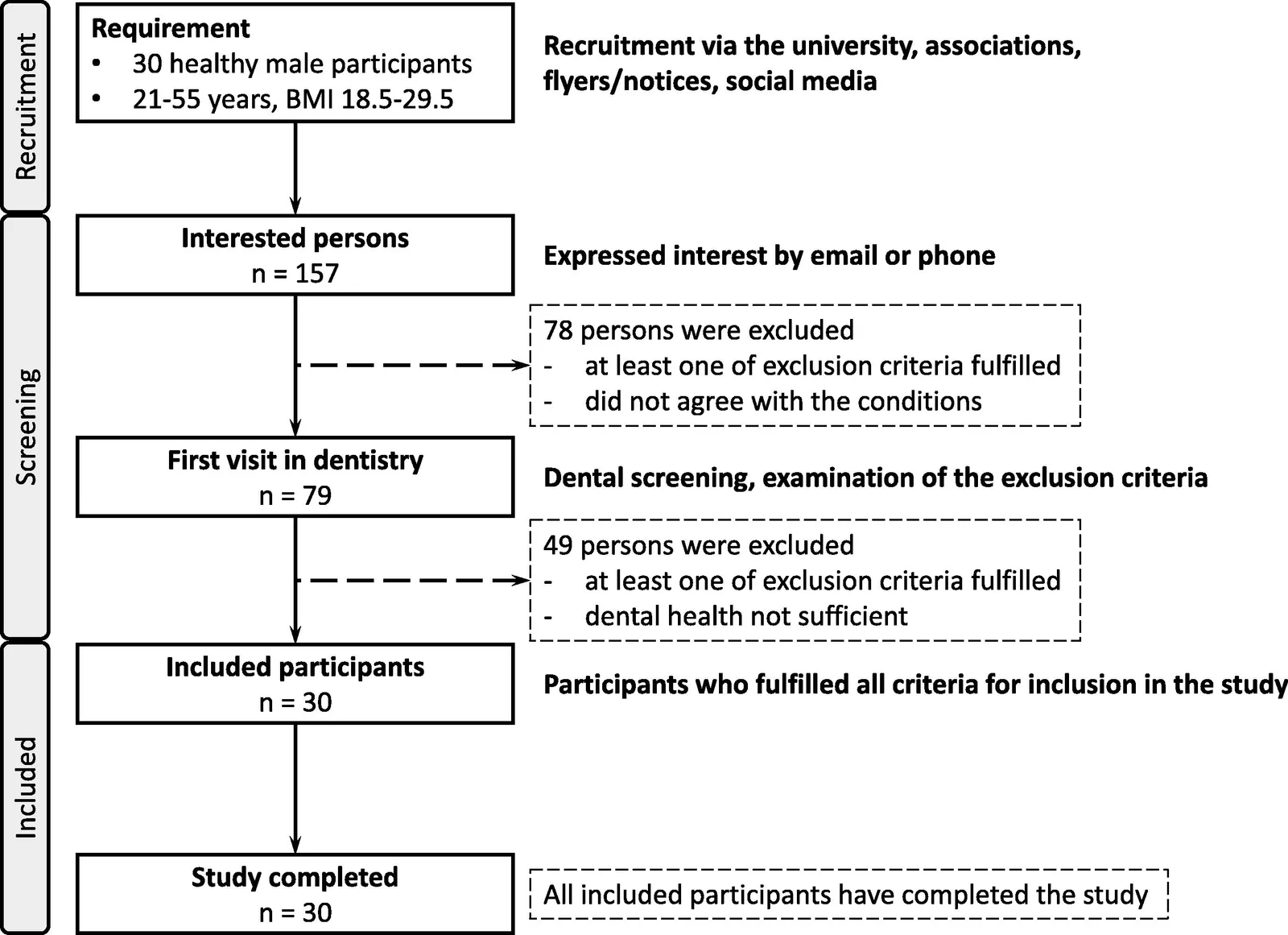
Flow diagram of the recruitment process and the screening factors. Recruitment process of the test participants, divided into the three phases of recruitment, screening and inclusion.

### Study procedure

Over the period of the study, participants underwent a total of eight visits (Fig. 2). The first visit was a screening visit to determine the eligibility of the participants (visit 1 [V1]). The impressions for the enamel appliance were also taken at this time. The second visit marked the actual start of the study (visit 2 [V2]). The third and sixth visits marked the start of the intervention, and the fifth and eight visits marked the end of the intervention (visit 3–8 [V3-8]). This structured sequence of visits ensured a systematic course of the study and allowed the effects of the interventions to be recorded in detail. Participants were randomized to receive either a phenolic compound-enriched apple juice-based drink (test drink) or a phenolic compound-free drink (placebo) after a 14-day wash-out period. The first 14-day intervention period was followed by another 14-day washout period. The participants then started the second intervention period with the other study beverage. Two fruit drinks were used as study drinks, which are comparable in composition to commercially available drinks. The test drink had a concentration of about 1.4 g/L phenolic compounds, the placebo contained almost no phenolic compounds. Table 1 shows an overview of the composition of the two study drinks. During the intervention, the participants consumed 750 mL of the study drink every day, distributed into 4 portions throughout the day. Participants consumed approximately 1 g of phenolic compounds per day from the study beverage. The study drinks were consumed at least one hour apart from other beverages and meals. Both the test and placebo drinks were filled into identical bottles and labelled with randomly assigned three-digit codes, in order to ensure that participants and investigators were blinded. An independent researcher generated the randomization sequence, and the study team responsible for enrolment and drink distribution had no access to the code assignment. Participants documented their daily food and drink intake using a customized web-based smartphone application. These records were used to monitor compliance with the low-phenolic diet and confirm that no significant dietary changes occurred during the study, thus minimizing the risk of dietary bias as a confounding factor.

**Fig. 2:**
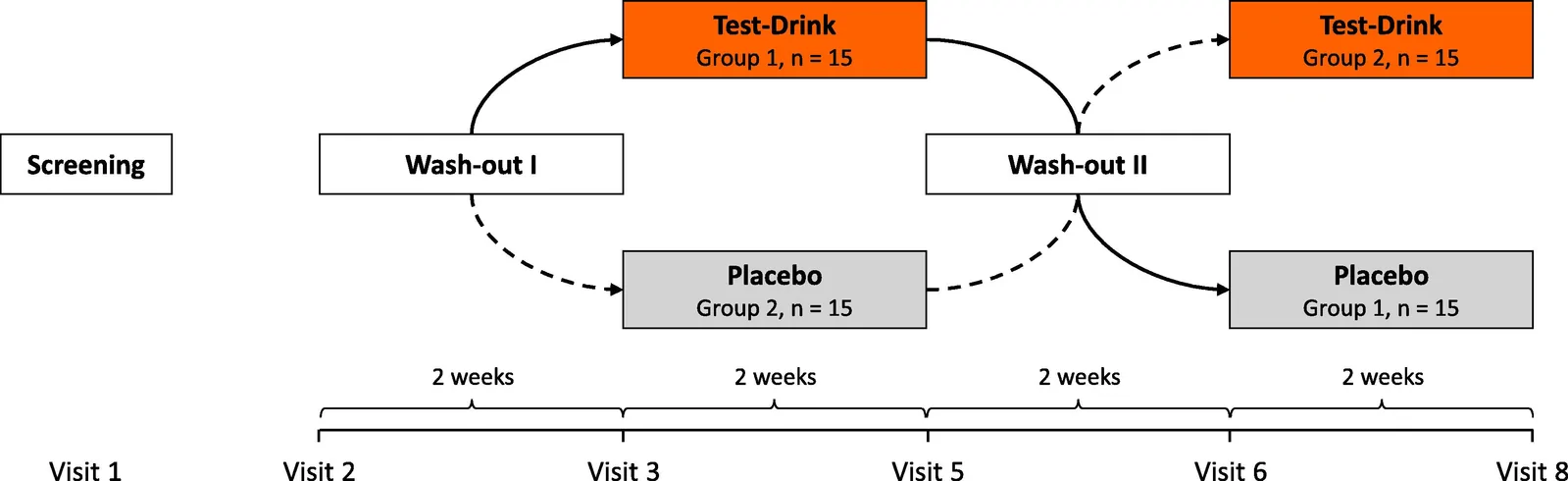
Study procedure, cross over design. Participants underwent two 2-week treatment periods (Test drink vs. placebo), each preceded by a 2-week washout period. Test drink = Phenolic compound-enriched apple juice-based drink, Placebo = Phenolic compound-Free Drink.

**Table 1:** Concentration of sugars, acids, phenolic compounds, as well as total acidity levels, and pH values in the test and placebo drinks.

	Test drink (enriched with phenolic compounds)	Placebo drink (depleted in phenolic compounds)
D-Glucose [g/L]	15.0	15.0
D-Fructose [g/L]	35.2	35.7
Sucrose [g/L]	2.9	3.7
Total sugars [g/L]	53.1	54.4
L-Malic acid [g/L]	4.5	4.6
Citric acid [g/L]	0.1	0.1
pH value [−]	3.2	3.3
Total acidity [g/L]	3.8	3.6
Total phenolic compounds(Folin-Ciocalteu) [g/L]	1.4	>0.1
Flavan-3-ols [mg/L]	138.5	n. d.
Dihydrochalcones [mg/L]	162.9	n. d.
Hydroxycinnamic acids [mg/L]	389.7	n. d.
Flavonols [mg/L]	15.9	n. d.
Total phenolic compounds(RP-UHPLC and HILIC) [mg/L]	707.0	n. d.

n. d.: not detected.

### Enamel appliance

To investigate the influence of phenolic compounds on dental health, a dental appliance was made individually for each participant (Fig. 3a). Caries progression during intervention phases I and II was quantified using two bovine tooth enamel samples fixed in a removable appliance (Fig. 3b). Participants wore the intraoral appliance continuously throughout the two-week intervention period, only removing it during mealtimes and for their daily oral hygiene routines. For the fabrication of the appliances, impressions of the participants upper and lower jaws were taken with alginate substitutes (Xanatasil, Kulzer GmbH, Hanau) and corresponding plaster models were fabricated upon enrollment of the participants (1st visit). Bovine incisors stored in 0.08 % thymol were used for the enamel samples. As all samples originated from a single tooth and were prepared to have identical size and surface characteristics. The samples were cleaned, sterilized with ethylene dioxide at 55 °C and gas sterilized. Any remaining ethylene dioxide components were washed out by storing them in sterile saline solution for 3 days. This was followed by preparation of blocks (5 × 3.5 × 3 mm) (Exakt 300; Exakt Apparatebau, Norderstedt, Germany) and embedding in epoxy resin (Technovit 4071; Heraeus Kulzer, Hanau, Germany) as well as preparation of a flat surface and polishing (grain size 4,000; silicon carbide, Phoenix Alpha; Wirtz-Bühler, Düsseldorf, Germany). The samples were demineralized with Buskes solution (pH 4.95; 7 days) to promote cariogenic progression during the planned 14-day procedure, and then partially coated with nail polish (control) [20]. This appliance was individually fabricated for each participant and new enamel samples were placed in each test phase. After the intervention, the bovine enamel samples were examined for cariogenic changes under the light microscope.

**Fig. 3:**
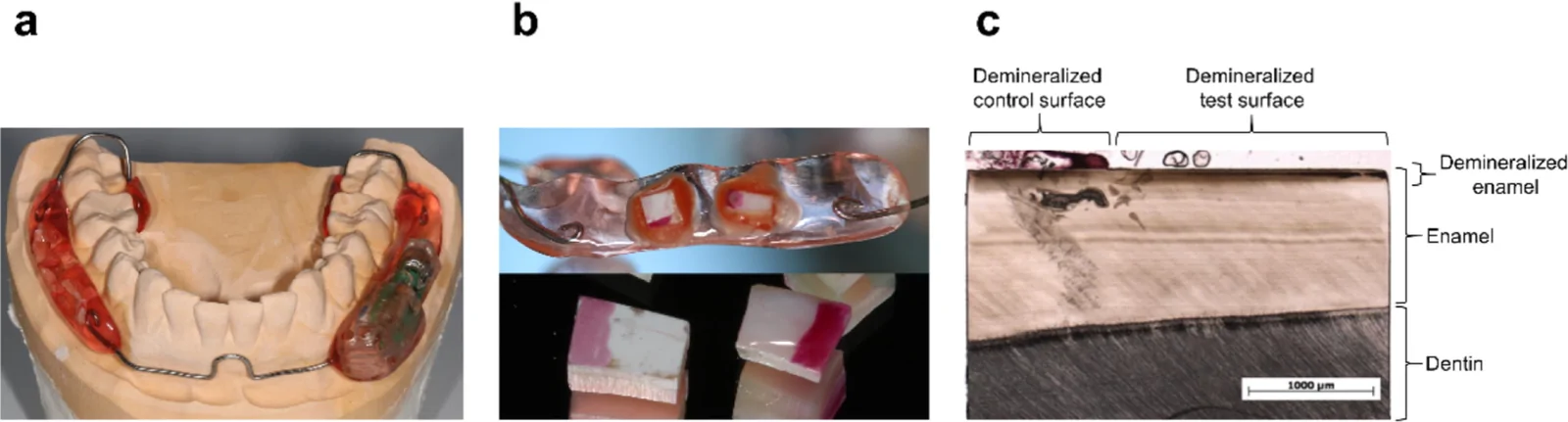
Enamel appliance. a. Enamel appliance for the analysis of influences on dental health; b. Bovine tooth enamel samples, in red, the sealed area of the control surface; c. Example of a microscopic analysis of dental enamel.

### Production of study drinks

The two study drinks either enriched with or depleted in phenolic compounds (test or placebo drink, resp.) were produced according to the production scheme depicted in the Supplementary Information (Fig. S1). In brief, a total of 400 kg apples (*cv*. Boskoop) were obtained from a local fruit producer (Dußling, Tailfingen, Germany). The apples were crushed by a progressing cavity pump with an extended compression casing with an integrated cutting mechanism (BTM, Seepex, Bottrop, Germany) and dejuiced using a horizontal filter press (HP-L 200, Bucher, Niederweningen, Schweiz), as outlined by Wagner et al. [21]. Subsequently, the raw apple juice was treated with a pectinase preparation (Fructozym P, Ebslöh, Geisenheim, Germany) at a concentration of 20 mL per t of apple juice for 1 h at approx. 15 °C. Coarse particles were then removed using a separator centrifuge (SAR3, GEA, Düsseldorf, Germany). Subsequently, the apple juice was rapidly heated to 78 °C with a heat tube exchanger (PAS1-PS2-81-V2, Mabo, Eppingen, Germany), hot-filled into 50 L kegs, and stored until further processing. In total, 350 L of apple juice were obtained.

To produce the test drink, 140 L of the apple juice were diluted with deionized water to a total sugar concentration of approximately 55 g/L. To reach the target total phenolic compound concentration of 1.4 g/L (determined by the Folin-Ciocalteu assay), a phenolic compound extract was produced from the remaining 210 L of apple juice and approximately 450 L of apple juice from concentrate. The phenolic concentration in the test drink was adjusted to 1.4 g/L, which corresponds to an intake of around 1 g per day based on a consumption of 750 mL. This concentration was chosen to represent a level of phenolic compound intake that is achievable from a nutritional perspective [22], while ensuring that the test drink would be the main source of phenolic compounds. The production of the latter was described in detail in the Supporting Information. The extraction of phenolic compounds was performed using a preconditioned adsorption column (PI 200/750 VOE-AB-FOG, Kronlab, Schermbeck, Germany) filled with 20 L of an adsorber resin (poly(styrene–divinylbenzene), Sepabeads SP70, Mitsubishi, Chiyoda, Japan; particle size > 250 µm). Applying aliquots of 110 L apple juice to the column, non-phenolic compounds were removed by subsequent flushing the column with 60 L of deionized water. Then, phenolic compounds were collected through elution with 25 L of ethanol (96 % v/v with 1 % methyl ethyl ketone, Martin Mundo, Mainz, Germany). The phenolic compound extract was then concentrated at 50 °C with a vacuum rotary evaporator (Laboranta 20 RC, Heidolph Scientific Instruments, Schwabach, Germany) and subsequently freeze-dried (Beta 2 8 LD plus, Martin Christ Gefriertrocknungsanlagen, Osterode, Germany) to prepare a powdery phenolic compound extract. Prior to the next extraction, the column was regenerated by flushing successively with 60 L deionized water, 20 L of 0.1 M sodium hydroxide (Tensid-Chemie, Muggensturm, Germany), 60 L deionized water, and 20 L of 0.1 M citric acid (Weifang Ensign Industry, Shandong, China). A total of 800 g of the obtained phenolic compound extract was added to the aforementioned diluted apple juice, yielding the test drink through vigorous mixing. The test drink was then rapidly heated to 78 °C using the previously mentioned heat-tube exchanger and hot-filled into 0.75 L amber glass bottles. The bottles were cooled to approximately 20 °C in a water bath and subsequently stored in a dark place at 20 °C.

Apple juice depleted of phenolic compounds was obtained as the eluate collected during the above-described phenolic compound extraction from apple juice, being used as the basis for the placebo drink. This juice depleted in phenolic compounds was diluted with deionized water to a total sugar concentration of approximately 55 g/L. To match the appearance of the test drink, artificial food dyes (4 mg/L Tartrazine, 8 mg/L Orange Yellow S, 1 mg/L Allura Red AC, 165 mg/L caramel color E150d; Döhler, Darmstadt, Germany) and a palm oil-based cloudifier emulsion (2 g/L; Döhler) were added. The placebo drink was then rapidly heated, hot-filled into 0.75 L amber glass bottles, and stored as described for the test drink.

### Determination of sugar, acid, and phenolic compound concentrations

The concentrations of D-glucose, D-fructose, sucrose, citric acid, and L-malic acid, as well as the total titratable acidity and pH value were measured as detailed by Dussling et al. [23].

The content of total phenolic compounds was determined spectrophotometrically using the Folin–Ciocalteu reagent with catechin as the reference compound [24]. Detailed identification and quantification of individual phenolic compounds and flavan-3-ol fractions were performed using the same sample preparation, UHPLC system, and methods for RP-UHPLC and HILIC, both coupled to DAD-FLD and DAD-ESI(−)-QTOF-HR-MS/MS, as outlined by Dussling et al. [23].

### Light microscopic examinations of the bovine tooth enamel samples

The lesion depths of the bovine tooth enamel samples were measured by light microscopy. Four measurements were taken at 200 µm intervals on the treated surface of each enamel sample. Two lesion depth measurements were made on the control surface. The extent of the cariogenic lesion is the difference between the mean values of the treated and control surfaces. A positive difference indicates that demineralization was greater on the test surface than on the control surface, indicating increased caries development. A negative difference means that demineralization was greater on the control surface, indicating remineralization on the test surface. As light microscopy required the samples to be cross-sectioned, a few specimens were lost or damaged during the subsequent preparation for scanning electron microscopy. Therefore, SEM analysis was only performed on approximately 80 % of the original samples. SEM was used to verify the lesion-depth patterns observed by light microscopy, but it was not used for statistical evaluation due to the smaller sample size.

### Oral parameters

At each visit, the study participants underwent a quick dental check-up, i.e., an assessment of the need for dental care (carious defects, mucosal lesions, etc.), a periodontal screening index (PSI), a plaque index according to the Quigley and Hein plaque index modified according to Turesky, the salivary flow rate and buffer capacity and a gingivitis index according to Löe and Silness [25], [26]. The flow rate of saliva is recorded as stimulated salivary flow. The participants had to chew a paraffin block for 1 min before the measurement and for 1 min during the saliva collection phase. To obtain the unstimulated saliva, the participant collects 5 ml in the resting position, i.e., without active stimulation of saliva production. The buffering capacity of saliva was measured with unstimulated saliva according to the method of Krasse (1986), modified according to Dodds et al. [27]. The plaque index according to Quigley and Hein modified according to Turesky is determined by staining the oral biofilm on six tooth surfaces (distobuccal, mid buccal, mesiobuccal, distolingual, mid lingual, mesiobuccal) with a staining solution (Mira-2-Ton, Hager Werken GmbH und Co. KG, Duisburg).

### Sample collection

Blood samples were taken from each participant at the beginning (V3 and 6) and end (V5 and 8) of the two interventions. The various blood parameters listed in Table S2 were analyzed at the central laboratory of the UKSH in Kiel on the day of the survey. Blood samples for the central laboratory analysis were stored at 4 °C until transport. Participants did not report any adverse events throughout the study period.

### Statistics and Reproducibility

The statistical analysis and visualization of the data was carried out with the software R (Version 4.3.1.). The most important packages for the analysis of the data were “lme4”(Version 1.1–35.1), “lsmeans” (Version 2.30–0), “car” (Version 3.1–2), “corplot” (Version 0.92), and “corrgram” (Version 1.14). For the statistical analysis of the blood analysis data and oral parameters, either the mean and standard deviation were calculated according to the distribution of the data and a paired *t*-test was performed between the groups, or the median and the interquartile range were calculated and a paired Wilcoxon signed-rank test was performed. To account for the complexity of the oral cavity, the data evaluation started with the definition of an appropriate statistical mixed model [28]. The model includes the fixed factors treatment (test drink, placebo), the covariates saliva flow rate, saliva buffer capacity, saliva pH value, plaque index, gingivitis index and decay missing filled teeth (DMFT), the two-fold interaction effects and all three-fold interactions effects including the treatment factor. Further, random effects were included covering the participant (“id”), to capture inter-individual differences, and phase (“phase”) of the crossover design, which determines whether participants started with treatment or placebo. This analysis was not prespecified in the protocol but added post hoc based on data structure. The residuals were assumed to be normally distributed and to be homoscedastic. These assumptions are based on a graphical residual analysis. Based on this model, an analysis of variances (ANOVA) was conducted, followed by multiple contrast test [29] in order to compare the placebo and the test drink group. None of the participants dropped out of the study. A small number of missing values occurred due to technical issues during sample preparation and measurement. In these instances, only the affected study phase of each participant was excluded. This resulted in a total of 55 valid observations for the final analysis. As mixed-effect models cannot handle missing values, but are robust to unbalanced data, the analysis was performed on a per-protocol basis. The sample size was based on a previous study [30]. Assuming a conservative effect size of 0.6 (11 μm difference, SD = 18 μm [31]), a power of 80 %, and α = 0.05, 26 participants were required. 30 participants were enrolled to account for potential dropouts.

## Results

To study the effect of phenolic compounds, 30 healthy men (Table S2) received a phenolic compound-enriched apple juice-based drink (test drink, 1.4 g phenolic compounds per liter, Table 1), i.e., approx. 1.0 g phenolic compounds per day, or a placebo drink for two weeks in a double blinded study using a cross-over design. As a placebo drink the participants received an apple juice drink where only the phenolic compounds were removed but acid and sugar content remained the same (placebo, Table 1). The cariogenic effects were tested using an appliance with a bovine enamel inserted in the oral cavity of the participants (Fig. 3).

### Study drinks

Both the test and placebo drinks had similar total sugar concentrations, ranging from 53 to 54 g/L, and total acidities ranging from 3.8 to 3.6 g/L, respectively (Table 1). The total phenolic compound concentrations of the test and placebo drinks, as determined by the Folin–Ciocalteu assay, were 1.4 g/L and below 0.1 g/L, respectively. In the test drink, six flavan-3-ols, five hydroxycinnamic acids, four dihydrochalcones, five flavonols, and five oligomeric flavan-3-ol fractions with degrees of polymerization ranging from 3 to 7 were identified (Supporting Information Table S1). As the phenolic profile, along with data obtained from RP-UHPLC and HILIC-DAD-ESI(−)-QTOF-HR-MS/MS analyses (data not shown), closely matched those previously reported for apple juice of the same variety, their identification was not further discussed herein. Quantification of phenolic compounds using RP-UHPLC and HILIC-DAD-FLD yielded a total concentration of 707 mg/L in the test drink, which fell within the range of 154–1015 mg/L reported for 100 % apple juice [9], [23], [32]. In contrast, the phenolic compounds of the placebo drink were below the limit of detection (>0.5 mg/L) of the RP-UHPLC and HILIC-DAD-FLD methods.

### Clinical characteristics

Table S2 shows the clinical characteristics of the participants. The level of 1.0 g phenolic compounds per day is considered to be safe and can also be achieved through a phenol-rich diet [33]. Also, acute toxicity is excluded as this has been observed in animal studies at doses of 0.5–2 g/kg body weight [34]. A total of 115 of the 120 blood counts taken over the period of the study were available. The blood analyses show that the treatment had no negative influence on the blood parameters tested. CRP and IL-6 concentrations were below the detection limit in the majority of participants. In these cases, the next lower value was given (for CRP: 0.59, for IL-6: 3.49).

### Oral health status

Several oral parameters were recorded for each participant at the beginning of the study (V2) and at the start (visits 3 and 6) and at the end (visits 5 and 8) of each intervention. They are listed and described in Table 2. Parameters analyzed included salivary flow rate, salivary buffer capacity, salivary pH and dental health indices including plaque index, gingivitis index and DMFT index. No significant changes in these parameters were observed during the intervention. In total 55 examinations were fully completed.

**Table 2:** Recorded oral parameters and measured lesion depths of participants.

Oral parameters	Test drink (Phenolic compound-enriched apple juice-based drink)			Placebo (Phenolic compound-free drink)		
	Baseline n = 30^a^	After 2 weeks n = 26^a^	Adj. p-value^b^	Baseline n = 30^a^	After 2 weeks n = 29^a^	Adj. p-value^b^
Lesion depth (µm)	−	−2.56 (24.00)	−	−	−2.87 (18.6)	−
Saliva flow rate (mL/min)	2.51 (1.28)	2.41 (1.35)	0.92	2.28 (1.26)	2.61 (1.59)	0.19
Saliva buffer capacity (mmol/L/pH)	5.88 (1.00)	5.47 (0.95)	0.44	5.66 (1.28)	5.31 (1.22)	0.19
Saliva pH value	7.77 (0.25)	7.75 (0.33)	0.92	7.82 (0.25)	7.73 (0.29)	0.19
Plaque Index	1.88 (0.68)	2.07 (0.61)	0.70	2.01 (0.54)	2.18 (0.68)	0.19
Gingivitis Index	0.62 (0.31)	0.70 (0.21)	0.72	0.6 (0.24)	0.71 (0.25)	0.19
DMFT	5.54 (4.85)	5.79 (4.89)	0.92	5.67 (4.75)	5.67 (4.75)	1.00

^a^Mean (SD), ^b^ paired t-Test, p-values adjusted using Benjamini-Hochberg correction, DMFT = decay missing filled teeth, Gingivitis Index (Ranges from 0 to 3).

### Enamel lesion depths

#### Light microscopy

In order to investigate the carious effects, the depths of the lesions in the bovine enamel samples were determined using light microscopy. The lesion depth in the bovine enamel was defined as the primary outcome parameter of the study; i.e., the difference between the test and control surfaces.

In some cases, the lesion was deeper on the treated surface than on the control surface (Fig. 4a), resulting in a positive difference. In other cases, the lesion was deeper on the control surface (Fig. 4b), resulting in a negative difference.

**Fig. 4:**
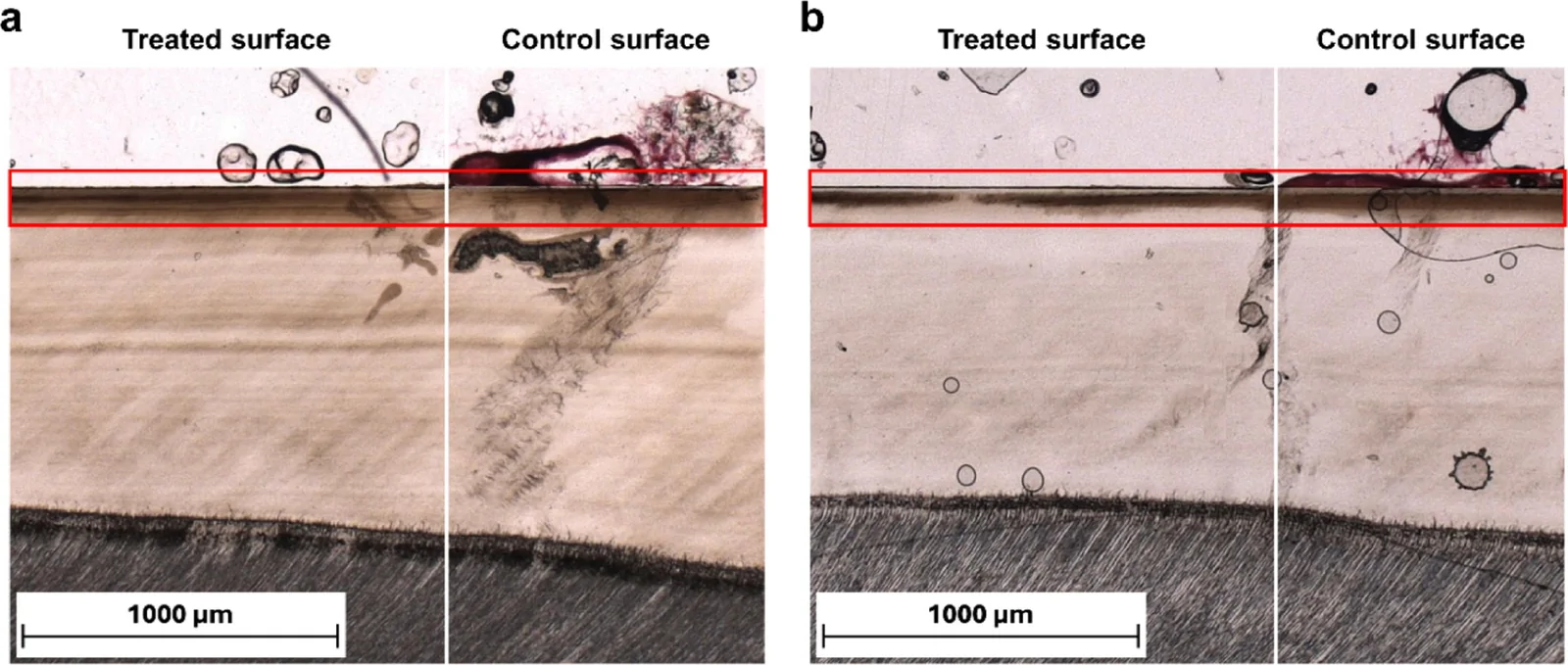
Light microscopic examination of the lesion depth of the treated and control surface. Lesion depths measured by light microscopy. a. Lesion depth on treated surface greater than control = lesion has increased in size, b. Lesion depth of the treated surface less than control = lesion has decreased in size.

The lesion depths caused after both interventions are shown in a histogram (Fig. 5). For the histogram the lesion depths were divided into 2.5 µm sections. If the lesion depth was negative, remineralization of the enamel was observed. As can be seen in the histogram, the lesion depths ranged from −55.99 to 52.94 µm. There were 27 positive cases indicating demineralization and 28 negative cases indicating remineralization. Clear differences were found at the extreme values. In the placebo group, there were eight cases with a change in lesion depth of > 10 µm, compared to six such cases in the treatment group. In contrast, for remineralization, the treatment group showed nine cases with changes <−10 µm compared to eight cases in the placebo group. Although there was a similar number of cases with changes of <−10 µm in both groups, the differences in the treatment group were more pronounced in this area. After consumption of the test drink the mean of the differences in lesions was −2.56 µm and 2.87 µm after consumption of the placebo drink. Statistical analysis by *t*-test, i.e., not taking into account the influence of other oral parameters (see 3.5 for statistical model with oral parameters), showed that the two mean differences in lesion depth were not significantly different from each other.

**Fig. 5:**
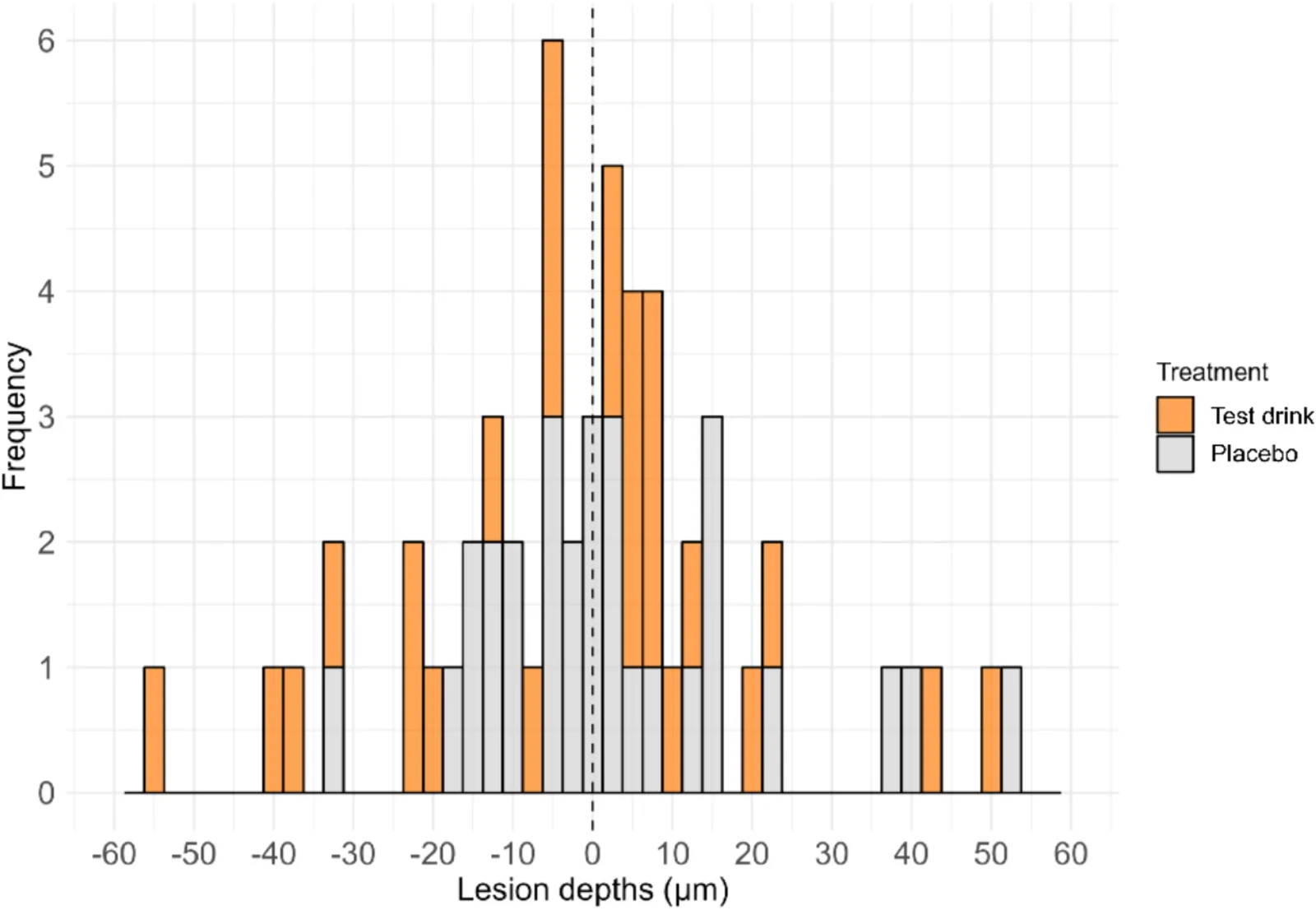
Histogram of the lesion depth distribution of the two treatments (Test drink and Placebo), Test drink = Phenolic compound-enriched apple juice-based drink, Placebo = Phenolic compound-free Drink. If the lesion depth was negative, the lesion in the treated surface was smaller than in the control area; if it was positive, it was larger. Raw data distributions are shown; statistical significance was determined by a mixed model (see Results of the mixed model).

#### Scanning electron microscopy (SEM)

In addition to the light microscopic analysis of the lesion depths in the enamel, we also performed a scanning electron microscopic (SEM, Fig. 6) examination for selected samples to substantiate the light microscopy results. They show similar lesion patterns that are consistent with the light microscopic observations and support our findings regarding differences in lesion depth between treatment groups.

**Fig. 6:**
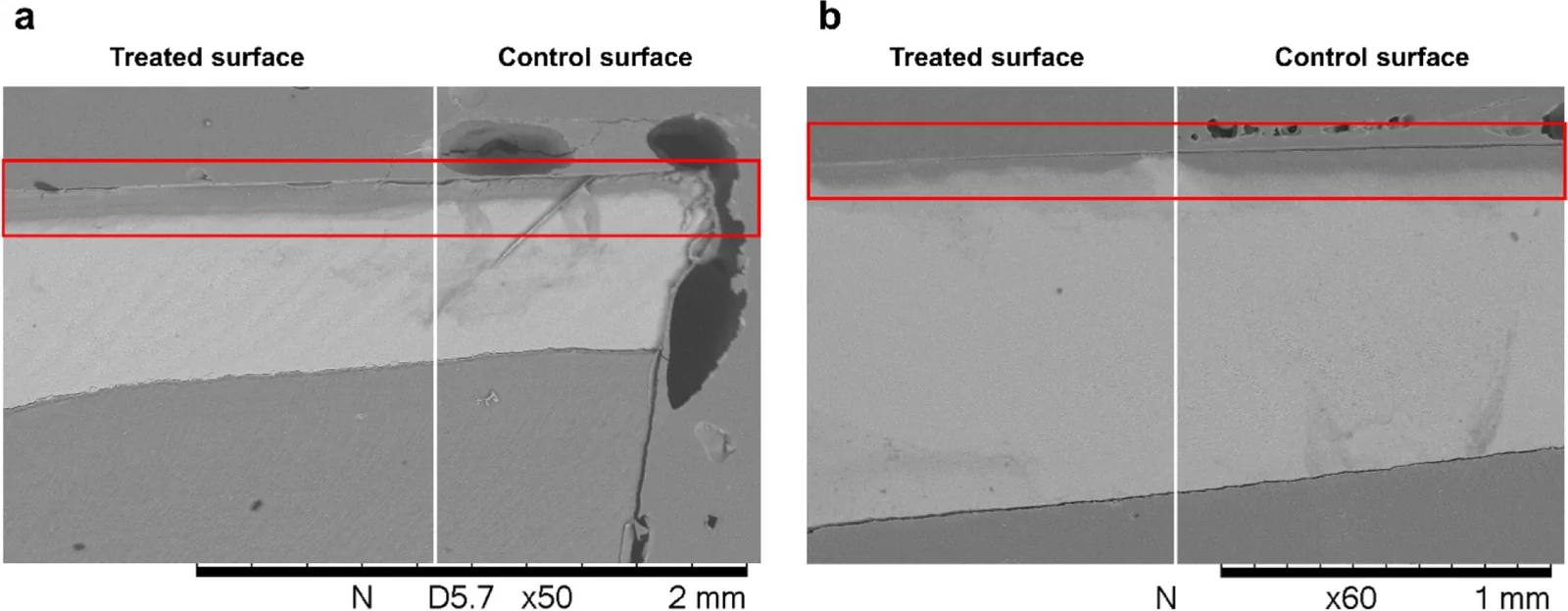
Scanning electron microscopic examination of the lesion depth of the treated and control surface. SEM was performed on approximately 80 % of the enamel samples and confirmed the same trend observed by light microscopy (mean lesion depth change by SEM: treatment = -0.94 µm; placebo = 4.47 µm). The figure shows a representative example. a. Lesion depth on treated surface greater than control = lesion has increased in size, b. Lesion depth of the treated surface less than control = lesion has decreased in size.

### Correlation between oral and dental health parameters

In order to evaluate the influence of various oral parameters, Pearson correlation coefficients were calculated (Fig. 7). The following parameters were included in the analysis: gingivitis index, plaque index, DMFT, salivary flow rate, salivary pH, salivary buffering capacity and lesion depth.

**Fig. 7:**
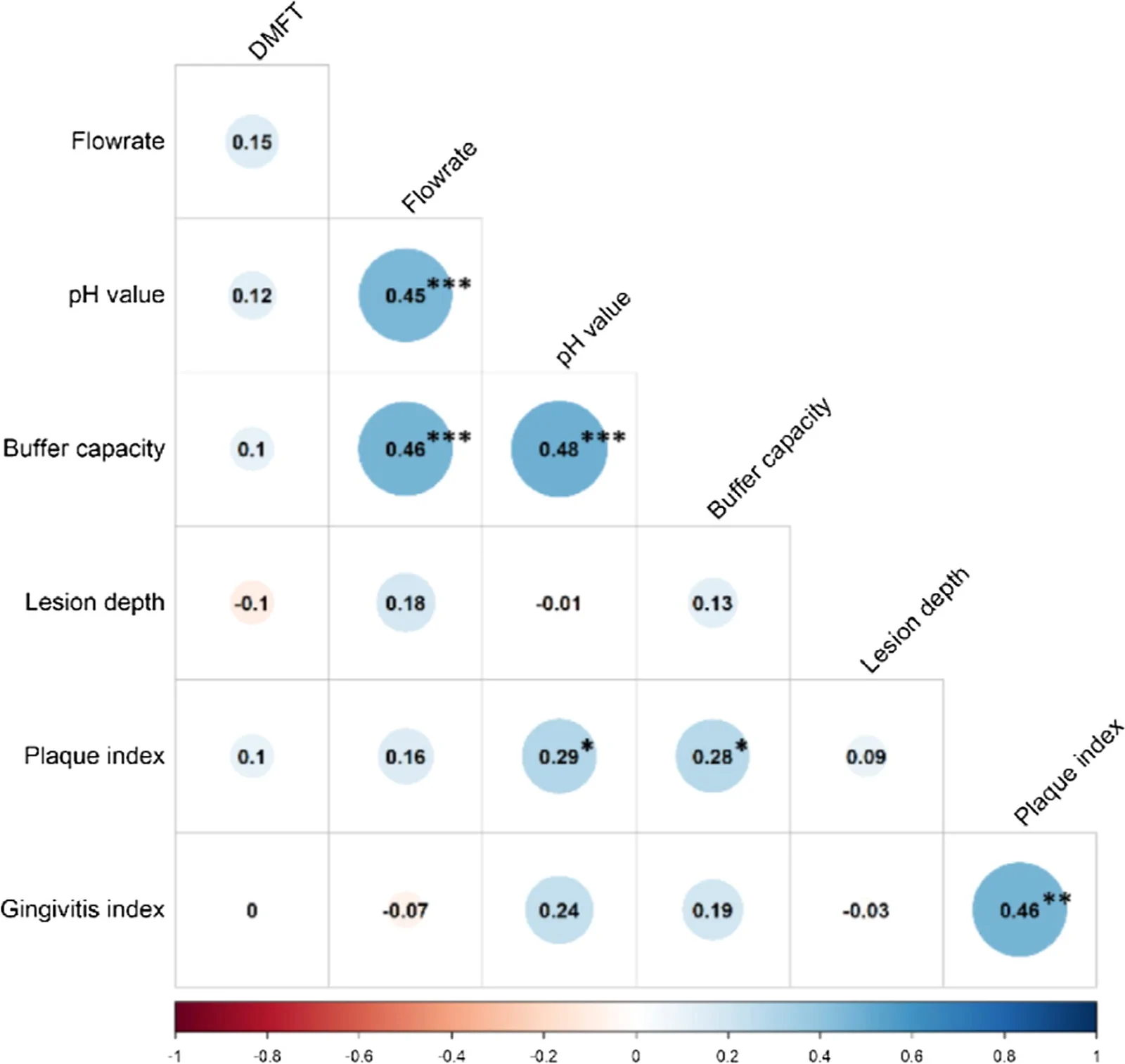
Pearsońs correlations between all measured oral health metrics The significance of Pearson's correlation coefficient is reported using the following convention: * p < 0.05, ** p < 0.01, *** p < 0.001. For the analysis, various dental parameters were recorded for each participant before and after the intervention. Buffer capacity = Buffer capacity of the unstimulated saliva, pH value = pH value of the unstimulated saliva, Flowrate = Flowrate of the stimulated saliva, DMFT = decay missing filled teeth, Lesion depth = lesion depth in bovine enamel.

Fig. 7 presents the associations between various oral health parameters. There was a significant positive association between plaque index and gingivitis index (r = 0.46, p-value = 0.0004), between plaque index and pH-value (r = 0.29, p-value = 0.0345), and between plaque index and buffer capacity (r = 0.28, p-value = 0.0357). For saliva parameters, significant positive correlations were also found between flowrate and pH value (r = 0.45, p-value = 0.0005), flowrate and buffer capacity (r = 0.46, p-value = 0.0004), and pH value and buffer capacity (r = 0.48, p-value = 0.0002).

### Results of the mixed model

To account for the complexity of the oral cavity in the analysis, a linear mixed model was defined that included not only the two treatments, but also important oral parameters and interactions (see 3.3). The detailed structure of this model is described in the “Materials and methods” section under “Statistical analyses”. All significant main effects and interactions that were identified performing an ANOVA.

Significant main effects of treatment, buffering capacity, salivary pH, plaque index and gingivitis index on lesion depth were found (Appendix see Table S3). However, these significant main effects are linked by interactions, that also have a significant influence on lesion depth. Therefore, in this analysis we focused specifically on the significant triple interaction terms (Table 3), as these provide a comprehensive representation of the relationships between the predictors and the dependent variable. By examining these interactions, we aimed to gain a detailed understanding of the underlying processes, while avoiding the risk of reporting only isolated main effects, the interpretation of which may be incomplete. The ANOVA revealed several significant effects on lesion depth. Some significant interactions were identified, indicating complex relationships among the predictors. In particular, the interactions “Treatment:Flowrate:Gingivits index”, “Treatment:Buffer capacity:DMFT”, “Treatment:Buffer capacity:Plaque index”, “Treatment:pH value:DMFT”, “Treatment:DMFT:Plaque index”, “Treatment:DMFT:Gingivits index” and “Treatment:Plaque index:Gingivits index” had a significant influence on the lesion depth in the enamel. The ANOVA analysis revealed that some triple interactions had a significant effect on lesion depth. Estimates for these significant interactions were then calculated and are shown in Table S4. It should be noted, however, that these estimates depend specifically on the combinations of other variables included in the model. The exact constellations and values of these additional variables that influence the calculated estimates cannot be directly identified. Therefore, the interpretation of these interaction effects requires careful consideration of the overall context of the model as well as the underlying data.

**Table 3:** ANOVA of the Model 1 all investigated three-way interaction terms of the model.

Variable/Effect	Chisq	p-value
Treatment: Flowrate: Buffer capacity	0.198	0.648
Treatment: Flowrate: pH value	0.989	0.309
Treatment: Flowrate: DMFT	0.475	0.492
Treatment: Flowrate: Plaque index	0.286	0.592
Treatment: Flowrate: Gingivits index	9.588	0.002 (**)
Treatment: Buffer capacity: pH value	0.896	0.342
Treatment: Buffer capacity: DMFT	35.118	<0.001 (***)
Treatment: Buffer capacity: Plaque index	5.049	0.020 (*)
Treatment: Buffer capacity: Gingivits index	0.225	0.638
Treatment: pH value: DMFT	16.552	<0.001 (***)
Treatment: pH value: Plaque index	0.003	0.953
Treatment: pH value: Gingivits index	0.458	0.516
Treatment: DMFT: Plaque index	5.193	0.019 (*)
Treatment: DMFT: Gingivits index	19.869	<0.001 (***)
Treatment: Plaque index: Gingivits index	9.346	0.002 (**)

Interaction terms from the ANOVA model. The colon (“:”) indicates interactions between the specified factors. Treatment = Test drink or Placebo, DMFT = decay missing filled teeth, Plaque Index [22], Gingivitis index [23], pH value = pH value of the unstimulated saliva, Buffer capacity = Buffer capacity of the unstimulated saliva, Flowrate = Flowrate of the stimulated saliva.

### Comparison of adjusted means between treatment groups

To examine the estimated effect of the treatment groups (test drink vs. placebo) on lesion depth a least-squares means analysis (LS-Means) was performed based on mixed model ANOVA which identified significant effects on the lesion depth variable.

The analysis of the LS-Means between the test drink and placebo groups resulted in an estimated difference of −55.7 µm with a standard error of 15.20. This difference is with a p-value of 0.019 statistically significant. This means that the adjusted mean lesion depth was less in the test drink compared to placebo treatment group. The adjusted means took into account the influences of the other variables and their interactions from the model. In addition, the confidence intervals limits for this difference ranged from a low of −96.8 µm to a high of −14.6 µm, still supporting a reduction in lesion depth in the test drink group.

To evaluate whether the effect depends on the participant’s dental health, an additional model (DMFT-Model, Table 5) was created that converted the continuous variable DMFT into a binary group variable. This allowed treatment outcomes to be analyzed separately for two defined groups: participants with healthier dental status (DMFT ≤ 5) and participants with less healthy dental status (DMFT > 5).

**Table 4:** Results for least squares means between the test drink and placebo.

Contrast	Estimate	Std. Error	Df	t-ratio	p-value	Lower CL	Upper CL
Test drink − Placebo	−55.70	15.20	4.29	−3.66	0.019	−96.8	−14.6

Test drink = Phenolic compound-enriched apple juice-based drink, Lower CL = Lower Confidence Limit, Upper CL = Upper Confidence Limit.

**Table 5:** Least squares mean results between the test drink and placebo in relation to the DMFT groups for caries risk (DMFT-Model).

Contrast	DMFT	Estimate	Std. Error	Df	t-ratio	p-value	Lower CL	Upper CL
Test drink-Placebo	<5	−55.38	17.34	4.12	−3.19	0.083	−123.39	12.63
Test drink-Placebo	≥5	−35.53	22.08	2.95	−1.61	0.329	−122.16	51.10

Comparison of LS-means between the treatment and placebo groups, divided into groups based on DMFT. Test drink = Phenolic compound-enriched apple juice-based drink, Lower CL = Lower Confidence Limit, Upper CL = Upper Confidence Limit, DMFT = decay missing filled teeth, Std. Error = standard errors, DF = degrees of freedom, DMFT = decay missing filled teeth.

Table 5 shows the results of the between-group effect comparisons for the DMFT model, in which the adjusted mean lesion depths of the two treatments were compared according to caries risk. Similar to Table 4, there was a consistent effect on lesion depth. In the group with low DMFT receiving the test drink, an estimated reduction in lesion depth of −55.38 µm was observed. The results show a statistical trend (p = 0.083), but do not reach the conventional level of significance. In the higher caries risk group (DMFT ≥ 5), a reduction in lesion depth was also estimated (estimate: −35.53 µm, p = 0.329). Although this effect was not statistically significant, a similar trend was observed.

## Discussion

In this study, we investigated the effects of phenolic compounds from apple on the balance of remineralization and demineralization of tooth enamel resulting in various lesion depths, and their potential benefits for dental health.

We found that consumption of the phenolic compound enriched drink was associated with reduced enamel lesion depth compared with the placebo drink. The estimated difference in adjusted mean lesion depth between the two treatments was −55.7 µm. This difference was statistically significant after adjusting for various oral factors (Table 4), as shown in the mixed model.

Light microscopy was selected as the primary method for the determination of the lesion depth because it enables consistent measurement across all samples, providing a robust foundation for statistical comparison between groups. To verify these results, scanning electron microscopy was performed on a subset of the same samples. SEM revealed the same pattern (the means were −0.94 µm for treatment group versus 4.47 µm for placebo group).

To the best of our knowledge, no human *in situ* or *in vivo* studies have evaluated the anticariogenic effects of apple phenolic compounds in the presence of fermentable sugars using enamel demineralization as functional endpoint. However, there have been *in vitro* studies investigating the potential anticariogenic effects of apple phenolic compounds [35]. Giacaman et. al demonstrated that apple juice concentrates can reduce sugar-induced demineralization of bovine enamel [35]. This result is consistent with our main findings. Although the controlled laboratory conditions provided valuable insights, they did not fully reflect the complexity of oral conditions and interactions. In contrast, our human intervention study allowed us to investigate the effect of phenolic compounds under real-life conditions, confirming similar beneficial effects. Our findings go beyond previous *in vitro* and animal studies and provide direct insights from a controlled human *in situ* model, thereby bridging the translational gap with high physiological relevance.

The observed shift towards remineralization under cariogenic conditions is consistent with mechanistic data showing that phenolic compounds can interfere with the key virulence factors of *S. mutans* and modulate host matrix interactions. For instance, flavonoids such as phloretin, a predominant phenolic compound found in apples, have been shown to inhibit *S. mutans* growth and formation. This effect is achieved by downregulating glycosyltransferase gene expression, thereby reducing the synthesis of extracellular polysaccharides. This compromises bacterial adhesion and reduces both acid production and acid tolerance [36]. In addition to microbial modulation, several cinnamic acid derivatives that are present in apples among other fruits (e.g., chlorogenic acid) can inhibit matrix metalloproteinase-3 (MMP3) involved in the degradation of mineralized dental tissues [37]. Inhibiting such enzymes could help stabilize the environment of the tooth surface and contribute to enamel maintenance. Although our study did not directly investigate these mechanisms, the consistency of our findings with those of previous mechanistic research strengths the relevance of phenolic compounds for dental health.

This mechanistic evidence is further supported by *in vivo* studies using animal models, which have shown that phenolic compounds exert a protective effect against dental caries. Treatment with proanthocyanidins significantly reduced the incidence of caries on smooth surface and resulted in less extensive carious lesion compared to the control group. There was reduction in caries on sulcal surfaces. A reduction in biofilm and insoluble glucans was also observed [38]. However, in comparison to our study, the procyanidin content of cranberries is generally much higher than that of apples [39]. Flavan-3-ols, including procyanidins accounted only for about 20 % of the total phenolic compounds in the phenol-rich drink in this study. The remaining were mainly hydroxycinnamic acids (55 %) and dihydrochalcones (23 %), while the smallest group was flavonols with only 2 % (Table S1). Although these phenolic compounds have not yet been shown to affect lesion depth or caries formation *in vivo*, some have demonstrated potential caries-inhibiting effects *in vitro* [36], [40].

In addition to demineralized enamel, we frequently observed a shift in the balance between demineralization and remineralization in favor of remineralization processes. In some cases, this shift led to a reduction in lesion depth in the treated samples. As the enamel samples were initially demineralized by acid treatment, such a shift in balance is reasonable. This effect has not been shown *in situ*, yet. It has already been observed that the enamel surface showed an increase in insoluble complexes after treatment with phenolic compound-rich grape seed extract, indicating remineralization of the enamel [7]. Xu et al. also found that phenolic compounds from Turkish gall extract promote the growth of hydroxyapatite crystals *in vitro* and *in vivo* by supporting the accumulation of calcium ions, thus enabling the formation of new mineralized crystal structures [41]. Our results confirm that phenolic compounds could potentially have a protective effect on demineralized enamel by shifting the balance in favor of remineralization.

As caries is a multifactorial disease, the effect of phenolic compounds were statistically analyzed using a complex mathematical model, which incorporates a number of important dental and oral factors as reviewed by Pitts et al. [2]. We included plaque levels, gingival inflammation, general caries status, and various salivary parameters. These oral parameters are known to influence the mineralization balance. Carvalho et al. demonstrated that the pattern of occlusal caries lesions depends on the amount of plaque [42]. Nazário et al. found a positive correlation between periodontal disease and root caries [43]. Saliva also plays a central role in the development of caries, with factors such as salivary pH, buffering capacity and flow rate influencing caries formation [44]. In addition to the main effects, all possible two-way interactions were included in the model as they may influence each other. This is supported by the observed Pearson correlations between key parameters such as the plaque index with the gingivitis index, pH value and buffer capacity as well as the relationships between flow rate, pH value and buffer capacity. These results highlight the complex interactions between oral parameters, which require more comprehensive, multivariate approaches to be fully understood. The correlations found in our study are supported by previous studies [45], [46], [47]. Alkhateeb et al. found a significant positive correlation between unstimulated salivary pH and buffering capacity [45]. A positive correlation between salivary pH and salivary flow rate has also been reported [46]. The association between plaque development and gingival inflammation is also well known [47]. It is also interesting to note that our data showed a slight but positive correlation between salivary pH and plaque index and buffering capacity (Fig. 7). To the best of our knowledge, there are no comparable studies to this effect. Given these complex relationships, we extended the analysis of our data to include interaction terms. In addition to two-way interactions, all three-way interactions involving treatment were included in our model. This enabled us to examine the interaction between treatment and other oral parameters and to identify and capture complex multidimensional relationships. Several three-way interactions involving treatment were statistically significant, indicating that the treatment effect was modified by specific combinations of oral characteristics. For instance, notable interaction terms included Treatment:Buffer capacity:DMFT, Treatment:pH value:DMFT, Treatment:DMFT:Gingivitis index.

To further investigate this complex interaction, subgroup comparisons between treatment groups were conducted using least squares means, extracted from the mixed model. We used subgroup comparisons that require categorical variables, thus participant’s DMFT scores were converted into a binary variable. The participants were divided into the groups “high caries risk” (DMFT > 5) and “low caries risk” (DMFT < 5). This approach is commonly used in correlational studies to analyze factors that may influence caries development [48], [49]. This approach allowed us to deeper evaluate the interaction with participants DMFT status by further analyzing the adjusted means of these subgroups. The results indicated that treatment with the test drink resulted in a reduction in lesions in both DMFT groups, with a particularly notable effect observed in the low caries risk group (DMFT < 5). In this group, lesions were smaller, with an estimated difference of −55.38 µm (Table 5), showing a clear, yet not significant, trend (p = 0.08). In contrast, the effect in the high caries risk group was smaller, with an estimated difference of −35.53 µm (Table 5). These results suggest that, on average, participants with lower DMFT scores had smaller lesions when using the test drink and tended to benefit more from it than those with poorer dental health. Our study is supported by Shetty et al. who identified differences in various oral parameters grouped by DMFT scores [49]. In addition, significant differences in salivary microbial communities were found between the different DMFT groups [48].

Additionally, ANOVA identified other significant three-way interactions that affected lesion depth in the model. These interactions indicate that the effects of the treatment cannot be considered in isolation, rather, they strongly influenced by the other variables. LS-Means comparisons use adjusted means to account for complex interactions between variables, helping to minimize potential biases from interactions and other confounding factors. This approach allows for a more accurate assessment of the differences between test drink and placebo drink.

The present study was conducted with a group of healthy male participants to ensure a homogeneous study population and minimize variability in such a small cohort. Although this approach strengthened the study’s robustness, it limits its generalizability. Future studies should therefore extend these findings to female participants and populations at higher risk of caries.

## Conclusion

Our study demonstrates the anticariogenic effects of phenolic compounds in the presence of sugar using a placebo-controlled, cross-over, *in situ* design with high physiological relevance. The present study therefore aims to bridge the translational gap between simplified *in vitro* studies and real-life consumption. Unlike previous studies, which primarily examined early bacterial adherence or biofilm parameters, our approach quantifies functional mineral changes in enamel lesions under conditions that simulate real-life consumption. These findings emphasize that dietary phenolic compounds can influence the mineralization balance of enamel under common consumption conditions. As the caries-protective effects of phenolic compounds depend heavily on individual oral parameters, these variables were incorporated into the statistical analyses. This approach allowed us to consider the interplay of multiple factors contributing to the multifactorial nature of caries progression. A trend towards a more pronounced beneficial effect of the test drink was observed in individuals with lower DMFT scores, which are indicative of healthier dental status. However, this difference did not reach statistical significance. Further studies should include a more diverse and representative cohort, broader range of genders, ages and baseline oral health statuses to enhance the generalizability of findings on the health-modulating potential of dietary phenolic compounds.

## Ethical statement

This study was performed in line with the principles of the Declaration of Helsinki. Approval was granted by the Ethics Committee of the Department of Medicine of Kiel University (AZ.: D 546/21). All participants provided written informed consent following comprehensive information about the study procedures, as outlined in a participant information sheet. This study has been registered with the German Register of Clinical Studies (DRKS) under the registration number ID: DRKS00026713 (https://drks.de/search/de/trial/DRKS00026713).

## Funding sources

This work was financially supported by the German Federation of Industrial Research Associations (AiF) as part of the Industrial Collective Research (IGF) by the Federal Ministry for Economic Affairs and Climate Action based on a resolution of the German Parliament as part of the IGF project AiF 20741 N of the Research Association of the German Food Industry.

## Declaration of competing interest

The authors declare that they have no known competing financial interests or personal relationships that could have appeared to influence the work reported in this paper.

## Data Availability

The data supporting the findings of this study are available within the article and its Supplementary Information. Additional datasets analyzed during the study as well as the R code used for data processing and statistical analyses, are available from the corresponding author upon reasonable request.
