# Supplementary material for: Apple phenolic compounds enhance dental remineralization in human: a multifactorial in situ study
Source: J Adv Res. 2026 Jan 16;88:677–88. doi: 10.1016/j.jare.2026.01.034 (PMC13669607; doi:10.1016/j.jare.2026.01.034)
Supplement: Supplementary file 1 [file mmc1.pdf]

1 Supplementary information

Production of the apple juice concentrate

To produce apple juice from concentrate, three batches of apple juices were made, combined and concentrated as described in the following.

First, a total of 960 kg of apples (Malus domestica Borkh. cv. Boskoop) were sourced from a local fruit producer (Dußling, Tailfingen, Germany). An aliquot of 400 kg was crushed using a nitrogen-flushed mill (MultiCut, VaculiQ, Hamminkeln, Germany) with a continuous nitrogen flow of 3–5 L/min. The resulting mash was dejuiced using a spiral filter press (VaculiQ) equipped with a four-channel spiral and a 100 µm sieve, and an applied underpressure of 0.8 bar below atmospheric pressure. The raw juice was treated with 20 mL of pectinase preparation (Fructozym P, Erbslöh, Geisenheim, Germany) per ton of juice for 1 hour at approximately 15 °C. Coarse particles were removed using a separator centrifuge (SAR3, GEA, Düsseldorf, Germany). The juice was then rapidly heated to 78 °C using a heat tube exchanger (PAS1-PS2-81-V2, Mabo, Eppingen, Germany), hot-filled into 50 L kegs, and stored at 20 °C until further processing. In total, 250 L of spiral filter-pressed apple juice were produced.

Another aliquot of 400 kg of apples were crushed using a progressing cavity pump with an extended compression casing and integrated cutting mechanism (BTM, Seepex, Bottrop, Germany). Dejuicing of the mash was performed with a decanter centrifuge (Z23-3, Flottweg, Vilsbiburg, Germany) operating at 5200 rpm (outer bowl) and a differential screw speed of 8.7 rpm, with a 150 mm weir plate. Pectinase treatment, coarse particle removal, and pasteurization were carried out as described above. Decanter processing yielded 200 L of apple juice.

Lastly, another aliquot of 160 kg of apples were crushed using the previously described progressing cavity pump (BTM, Seepex). The mash was then heated to 50 °C with a heat tube exchanger (Alfa Laval, Lund, Sweden) and treated with 100 mL of pectinase preparation (Fructozym P, Erbslöh) for 1 hour at 50 °C. Dejuicing was carried out using a rack and cloth press (HPP400, Wahler, Germany) at a maximum pressure of 50 bar. Particle separation and pasteurization followed the same procedure as described above. This process yielded 100 L of apple juice.

The juices were combined and concentrated from 14 °Brix to approximately 40 °Brix using a pilot rising film evaporator (PAV 103B, Unipectin Ingredients, Eschenz, Switzerland). During evaporation, the flow rate was kept at 80 L/h and the preheater temperature was maintained at 72 °C. The steam pressure and the pressure of the evaporator were set at 1.2 bar above and 0.9 bar below atmospheric pressure, respectively. This process yielded 190 kg of apple juice concentrate, which was rapidly heated to 78 °C using a heat tube exchanger (PAS1-PS2-81-V2, Mabo) and hot-filled into 50 L kegs. Prior to phenolic compound extraction, the concentrate was diluted with deionized water to approximately 16 °Brix.

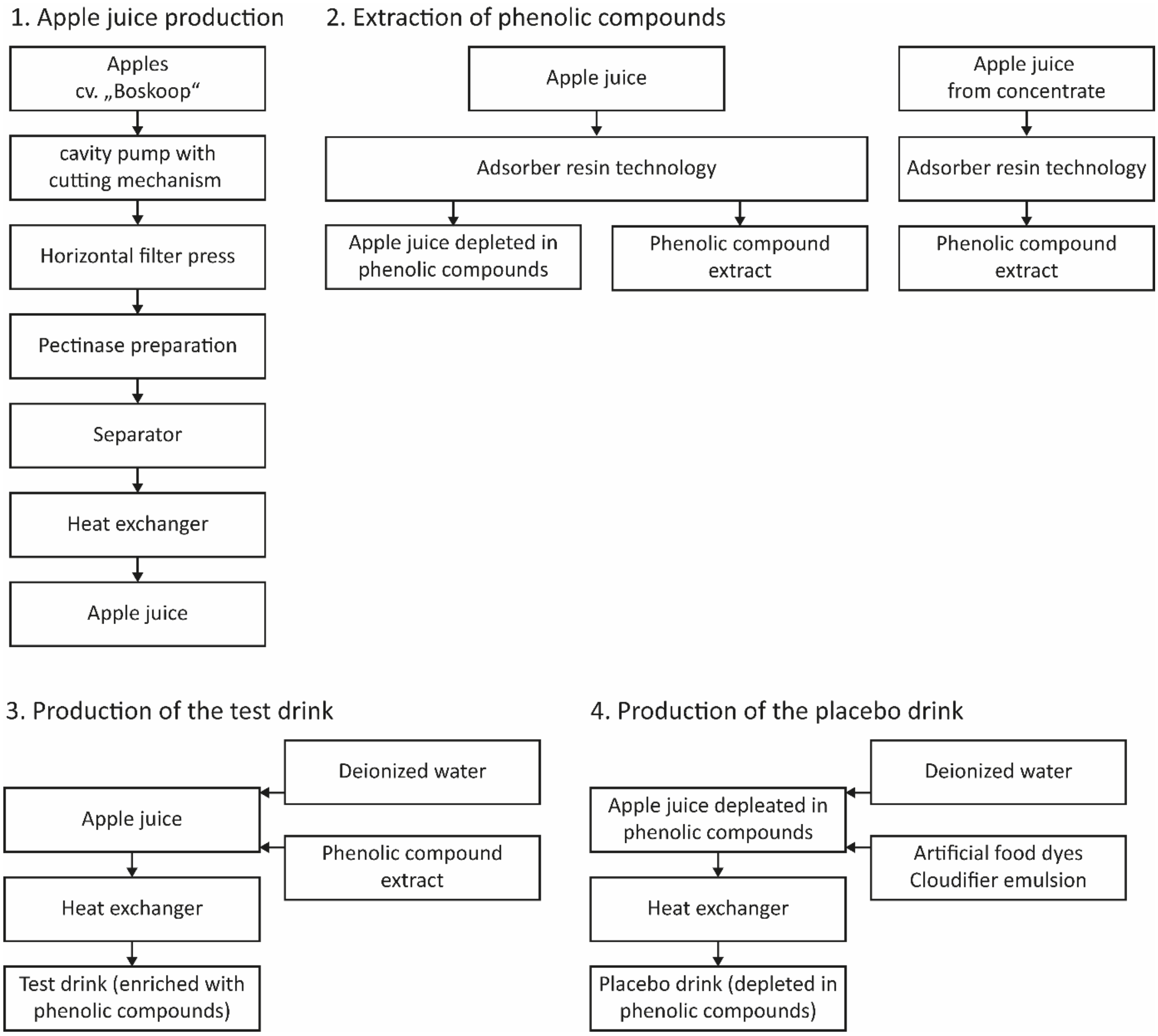

Fig. S1 Scheme for producing the (1) apple juice and (2) phenolic compound extract and apple juice depleted in phenolic compounds for the production of the (3) test drink (enriched with phenolic compounds) and the (4) placebo drink (depleted in phenolic compounds)

Table S1 Concentrations of phenolic compounds and procyanidin fractions in the Test drink and polyphenol-free drinks as measured by RP-UHPLC-DAD and HILIC-DAD

Phenolic compounds	Concentration [mg/L]	
	Test drink (enriched with phenolic compounds)	Placebo drink (depleted in phenolic compounds)
Flavan-3-ols		
Catechin	4.8	n. d.
Epicatechin	19.2	n. d.
Procyanidin B1	3.3	n. d.
Procyanidin B2	25.1	n. d.
Procyanidin dimer	0.9	n. d.
∑Procyanidin DP3	26.9	n. d.
Procyanidin C1	9.1	n. d.
∑Procyanidin DP4	19.3	n. d.
∑Procyanidin DP5	18.7	n. d.
∑Procyanidin DP6	12.0	n. d.
∑Procyanidin DP7	8.4	n. d.
**∑Flavan-3-ols**	138.5	n. d.
Dihydrochalcones		
3-Hydroxyphloretin 2'-*O*-xylosylglucoside	2.3	n. d.
3-Hydroxyphloretin 2'-*O*-glucoside	3.2	n. d.
Phloretin-2'-*O*-xyloglucoside	68.6	n. d.
Phloretin-2'-*O*-glucoside	88.7	n. d.
**∑Dihydrochalcones**	162.9	n. d.
Hydroxycinnamic acids		
p-Coumaroyl hexose	7.4	n. d.
4-*O*-caffeoylquinic acid	9.2	n. d.
5-*O*-caffeoylquinic acid	340.9	n. d.
Feruloyl hexose	2.6	n. d.
4-*O*-*p*-coumaroylquinic acid	29.6	n. d.
**∑Hydroxycinnamic acids**	389.7	n. d.
Flavonols		
Quercetin-3-*O*-galactoside	4.4	n. d.
Quercetin-3-*O*-glucoside	1.9	n. d.
Quercetin-3-*O*-xyloside	3.7	n. d.
Quercetin-3-*O*-arabinoside	2.4	n. d.
Quercetin-3-*O*-rhamnoside	3.6	n. d.
**∑Flavonols**	15.9	n. d.
**∑ Total phenolic compounds (RP-UHPLC / HILIC)**	707.0	n. d.

n. d.: not detected.

Table S2 Baseline and Post-Intervention Clinical Characteristics

Characteristics	Test drink (Phenolic compound-enriched apple juice-based drink)			Placebo (Phenolic compound-free drink)		
	Baseline[a]	After 2 weeks[a]	Adj. p-value[b]	Baseline[a]	After 2 weeks[a]	Adj. p-value[b]
Age (years)	28.5 (23.0, 34.0)					
BMI (kg/m^2)	24.7 (2.4)					
Leukocytes (10^9/l)	6.7 (3.2)	5.9 (1.2)	0.41	5.8 (1.2)	5.7 (2.0)	0.79
Erythrocytes (10^9/l)	5.1 (0.4)	5.1 (0.4)	0.63	5.1 (0.3)	5.0 (0.4)	0.30
Hemoglobin (g/dl)	15.1 (1.1)	15.0 (1.1)	0.35	15.2 (0.9)	14.9 (1.0)	0.17
Hematocrit (%)	42.7 (40.9, 44.0)	42.1 (40.3, 43.3)	0.48	42.7 (41.1, 44.3)	42.1 (40.2, 43.1)	0.11
MCV (fl)	83.8 (3.5)	83.4 (3.7)	0.41	83.8 (3.4)	83.3 (3.2)	0.31
MCH (pg)	29.8 (1.3)	29.6 (1.3)	0.40	29.7 (1.5)	29.6 (1.3)	0.51
MCHC (g/dl)	35.6 (1.0)	35.5 (1.0)	0.54	35.5 (1.1)	35.5 (1.1)	0.95
Thrombocytes (10^9/l)	264 (217, 290)	237 (225, 272)	0.48	248 (217, 281)	234 (221, 262)	0.46
MPV (fl)	10.4 (0.7)	10.4 (0.7)	0.54	10.6 (0.8)	10.5 (0.7)	0.49
Sodium (mmol/l)	139.9 (2.0)	139.7 (1.3)	0.54	140.1 (1.6)	139.9 (1.5)	0.57
Potassium (mmol/l)	4.1 (0.3)	4.2 (0.3)	0.22	4.2 (0.4)	4.1 (0.3)	0.31
Creatinine (µmol/l)	87.0 (16.5)	86.0 (13.8)	0.54	85.9 (15.1)	85.3 (14.8)	0.79
Glucose (mg/dl)	88.7 (6.5)	93.6 (15.0)	0.35	89.6 (10.5)	93.6 (13.6)	0.51
Triglycerides (mmol/l)	1.1 (0.8, 1.9)	1.1 (0.9, 1.4)	0.76	1.4 (1.1, 1.9)	1.2 (0.8, 1.4)	0.11
ALT (U/l)	22.6 (16.4,29.1)	18.0 (13.7, 29.1)	0.47	19.9 (12.0, 30.8)	19.2 (14.2, 30.4)	0.54
GGT (U/l)	25.5 (16.5,30.0)	26.0 (16.0, 39.0)	1.00	27.0 (18.0, 38.0)	26.0 (17.0, 37.0)	0.46
CRP (mg/l)	0.7 (0.6, 1.3)	0.6 (0.6, 1.1)	0.48	0.6 (0.6, 0.9)	0.6 (0.6, 1.0)	0.54
IL-6 (ng/l)	3.5 (3.5, 3.5)	3.5 (3.5, 3.5)	0.52	3.5 (3.5, 3.5)	3.5 (3.5, 3.5)	0.54
Tryptophan (µmol/l)	63.9 (7.9)	61.9 (6.9)	0.35	67.5 (10.6)	64.3 (11.6)	0.51

Data are presented as [a] Median (IQR) and Mean (SD). Statistical analysis was performed using a [b]paired t-test for normally distributed data or a paired Wilcoxon signed-rank test for non-normally distributed data. P-values were adjusted using th Benjamini-Hochberg correction. BMI = body mass index, ALT = alanine transaminase, GGT = gamma-glutamyltransferase, CRP = C-reactive protein, IL-6 = interleukin 6, MCV = mean cellular volume, MCH = mean cellular hemoglobin, MCHC = mean cellular hemoglobin concentration, MPV = mean platelet volume. The limit of detection for CRP was 0.6 mg/dl and for IL-6 3.5 ng/l

Table S3 Results of the mixed-model ANOVA (model 1)

Variable/ Effect	Chisq	Df	p-value
Treatment	119.9332	1	<0.0001
Flowrate	5.8373	1	0.0157
Buffer capacity	18.2537	1	<0.0001
pH value	9.2441	1	0.0024
DMFT	5.7330	1	0.0166
Plaque index	20.7071	1	<0.0001
Gingivitis index	170.1078	1	<0.0001
Treatment:Flowrate	5.3893	1	0.0203
Treatment:Buffer capacity	2.0057	1	0.1570
Treatment:pH value	11.1591	1	0.0008
Treatment:Plaque index	5.3417	1	0.0208
Treatment:Gingivitis index	18.5469	1	<0.0001
Treatment:DMFT	5.8531	1	0.0155
Flowrate:Buffer capacity	23.5787	1	<0.0001
Flowrate:pH value	6.1593	1	0.0131
Flowrate:DMFT	4.5019	1	0.0339
Flowrate:Plaque index	3.1743	1	0.0748
Flowrate:Gingivitis index	6.4220	1	0.0113
Buffer capacity:pH value	7.6829	1	0.0056
Buffer capacity:DMFT	0.0014	1	0.9700
Buffer capacity:Plaque index	13.2528	1	0.0003
Buffer capacity:Gingivitis index	14.5947	1	0.0001
pH value:DMFT	1.0546	1	0.3040
pH value:Plaque index	23.6986	1	<0.0001
pH value:Gingivitis index	4.0243	1	0.0449
DMFT:Plaque index	0.1746	1	0.6760
DMFT:Gingivitis index	2.3525	1	0.1250
Plaque index:Gingivitis index	0.2631	1	0.6080
Treatment:Flowrate:Buffer capacity	0.2090	1	0.6480
Treatment:Flowrate:pH value	1.0361	1	0.3090
Treatment:Flowrate:DMFT	0.4730	1	0.4920
Treatment:Flowrate:Plaque index	0.2875	1	0.5920
Treatment:Flowrate:Gingivitis index	9.6799	1	0.0019
Treatment:Buffer capacity:pH value	0.9018	1	0.3420
Treatment:Buffer capacity:DMFT	36.7060	1	<0.0001
Treatment:Buffer capacity:Plaque index	5.4105	1	0.0200
Treatment:Buffer capacity:Gingivitis index	0.2207	1	0.6380
Treatment:pH value:DMFT	16.8109	1	<0.0001
Treatment:pH value:Plaque index	0.0035	1	0.9530
Treatment:pH value:Gingivitis index	0.4212	1	0.5160
Treatment:DMFT:Plaque index	5.5351	1	0.0186
Treatment:DMFT:Gingivitis index	20.6508	1	<0.0001
Treatment:Plaque index:Gingivitis index	9.3041	1	0.0023

The table presents Chi-square values (Chisq), degrees of freedom (Df), and p-values for the fixed effects. The first column lists the examined variables, including main effects interaction terms.
Treatment = Test drink or Placebo, DMFT = decay missing filled teeth, Plaque Index (Turesky et al., 1970), Gingivitis index (LOE & SILNESS, 1963), pH value = pH value of the unstimulated saliva, Buffer capacity = Buffer capacity of the unstimulated saliva, Flowrate = Flowrate of the stimulated saliva

Table S4 Summary of the mixed-model analysis (model 1)

Effect	Estimate	Std. Error	df	t-value	p-value
Test drink	1398.939	1461.452	8.637	0.957	0.364
Placebo	-352.309	1121.954	7.100	-0.314	0.763
Test drink:Flowrate	101.411	215.374	8.830	0.471	0.649
Placebo:Flowrate	754.786	335.885	8.866	2.247	0.052
Test drink:Buffer capacity	-1011.304	433.253	9.219	-2.334	0.044
Placebo:Buffer capacity	-501.300	265.473	8.823	-1.888	0.092
Test drink:pH value	-88.843	210.710	8.577	-0.422	0.684
Placebo:pH value	59.170	159.338	7.203	0.371	0.721
Test drink:Plaque index	1886.187	559.785	8.148	3.369	0.010
Placebo:Plaque index	1654.190	517.786	7.149	3.195	0.015
Test drink:Gingivitis index	-1989.460	1647.201	8.782	-1.208	0.259
Placebo:Gingivitis index	-2454.772	1649.114	8.584	-1.489	0.172
Test drink:DMFT	207.012	59.164	7.931	3.499	0.008
Placebo:DMFT	-166.819	66.472	9.583	-2.510	0.032
Test drink:Flowrate:Buffer capacity	23.790	15.956	8.490	1.491	0.172
Placebo:Flowrate:Buffer capacity	15.405	4.453	8.266	3.460	0.008
Test drink:Flowrate:pH value	-43.954	35.578	8.287	-1.235	0.251
Placebo:Flowrate:pH value	-104.584	45.687	8.602	-2.289	0.049
Test drink:Flowrate:DMFT	1.675	1.458	9.778	1.148	0.278
Placebo:Flowrate:DMFT	3.179	1.651	6.373	1.925	0.100
Test drink:Flowrate:Plaque index	16.621	10.336	4.982	1.608	0.169
Placebo:Flowrate:Plaque index	6.826	12.887	8.945	0.530	0.609
Test drink:Flowrate:Gingivitis index	78.000	50.010	8.108	1.560	0.157
Placebo:Flowrate:Gingivitis index	-104.127	27.532	8.081	-3.782	0.005
Test drink:Buffer capacity:pH value	123.008	53.754	9.092	2.288	0.048
Placebo:Buffer capacity:pH value	59.774	35.916	9.154	1.664	0.130
Test drink:Buffer capacity:DMFT	12.446	3.390	5.992	3.671	0.010
Placebo:Buffer capacity:DMFT	-17.457	3.924	8.372	-4.449	0.002
Test drink:Buffer capacity:Plaque index	26.080	29.355	9.767	0.888	0.396
Placebo:Buffer capacity:Plaque index	92.624	21.954	8.013	4.219	0.003
Test drink:Buffer capacity: Gingivitis index	-90.495	49.713	9.985	-1.820	0.099
Placebo:Buffer capacity:Gingivitis index	-117.276	33.056	8.294	-3.548	0.007
Test drink:pH value:DMFT	-31.718	8.670	7.681	-3.658	0.007
Placebo:pH value:DMFT	28.335	10.349	9.691	2.738	0.021
Test drink:pH value:Plaque index	-280.977	88.285	8.851	-3.183	0.011
Placebo:pH value:Plaque index	-273.992	76.825	7.320	-3.566	0.008
Test drink:pH value:Gingivitis index	237.018	242.852	8.849	0.976	0.355
Placebo:pH value:Gingivitis index	441.679	225.493	8.676	1.959	0.083
Test drink:Plaque index:DMFT	-6.085	3.753	6.965	-1.621	0.149
Placebo:Plaque index:DMFT	5.356	4.245	8.380	1.262	0.241
Test drink:Gingivitis index:DMFT	-29.284	10.564	9.949	-2.772	0.020
Placebo:Gingivitis index:DMFT	27.615	7.420	1.730	3.722	0.081
Test drink:Plaque index:Gingivitis index	254.283	94.857	9.580	2.681	0.024
Placebo:Plaque index:Gingivitis index	-68.388	37.161	7.132	-1.840	0.108

The table presents the estimated coefficients (Estimate), standard errors (Std. Error), degrees of freedom (df), t-values, and p-values for the fixed effects. Treatment = Test drink or Placebo, DMFT = decay missing filled teeth, Plaque Index (Turesky et al., 1970), Gingivitis index (LOE & SILNESS, 1963), pH value = pH value of the unstimulated saliva, Buffer capacity = Buffer capacity of the unstimulated saliva, Flowrate = Flowrate of the stimulated saliva

Table S5 R packages used in the analysis

R-package	Description	Version
lme4	Linear Mixed-Effects Models	1.1-35.5
lsmeans	Least-Squares Means	2.30-0
emmeans	Estimated Marginal Means	1.10.6
multcomp	Simultaneous inference in general parametric models	1.4-26
car	Companion to applied regression	3.1-3
carData	Companion to applied regression data sets	3.0-5
corrgram	Plot a correlogram	1.14
corrplot	Visualization of a correlation matrix	0.95
dplyr	A grammar of data manipulation	1.1.4
ggplot2	Data Visualization using the grammar of graphics	3.5.1
RColorBrewer	ColorBrewer palettes	1.1-3

List of R packages used in the analysis, including package names, descriptions, and version numbers. All analyses were conducted in R version 4.3.1.
